# Transgenic CHD1L Expression in Mouse Induces Spontaneous Tumors

**DOI:** 10.1371/journal.pone.0006727

**Published:** 2009-08-24

**Authors:** Muhan Chen, Jian-dong Huang, Liang Hu, Bo-jian Zheng, Leilei Chen, Sze Lan Tsang, Xin-yuan Guan

**Affiliations:** 1 Department of Clinical Oncology, The University of Hong Kong, Pokfulam, Hong Kong, China; 2 Department of Biochemistry, The University of Hong Kong, Pokfulam, Hong Kong, China; 3 Department of Microbiology, The University of Hong Kong, Pokfulam, Hong Kong, China; 4 State Key Laboratory of Oncology in Southern China, Cancer Center, Sun Yat-sen University, Guangzhou, China; University of Texas MD Anderson Cancer Center, United States of America

## Abstract

**Background:**

Amplification of 1q21 is the most frequent genetic alteration in hepatocellular carcinoma (HCC), which was detected in 58–78% of primary HCC cases by comparative genomic hybridization (CGH). Using chromosome microdissection/hybrid selection approach we recently isolated a candidate oncogene *CHD1L* from 1q21 region. Our previous study has demonstrated that *CHD1L* had strong oncogenic ability, which could be effectively suppressed by siRNA against *CHD1L*. The molecular mechanism of *CHD1L* in tumorigenesis has been associated with its role in promoting cell proliferation.

**Methodology/Principal Findings:**

To further investigate the *in vivo* oncogenic role of *CHD1L*, *CHD1L* ubiquitous-expression transgenic mouse model was generated. Spontaneous tumor formations were found in 10/41 (24.4%) transgenic mice, including 4 HCCs, but not in their 39 wild-type littermates. In addition, alcohol intoxication was used to induce hepatocyte pathological lesions and results found that overexpression of CHD1L in hepatocytes could promote tumor susceptibility in *CHD1L*-transgenic mice. To address the mechanism of *CHD1L* in promoting cell proliferation, DNA content between *CHD1L*-transgenic and wildtype mouse embryo fibroblasts (MEFs) was compared by flow cytometry. Flow cytometry results found that *CHD1L* could facilitate DNA synthesis and G1/S transition through the up-regulation of Cyclin A, Cyclin D1, Cyclin E, CDK2, and CDK4, and down-regulation of Rb, p27^Kip1^, and p53.

**Conclusion/Significance:**

Taken together, our data strongly support that *CHD1L* is a novel oncogene and plays an important role in HCC pathogenesis

## Introduction

Hepatocellular carcinoma (HCC) is one of the most common solid tumors in the world affecting one million individuals annually [1]. The prognosis of HCC is very poor and the overall 5-year survival rate is less than 5%, mainly because of the late diagnosis [2]. Although different etiologic factors such as hepatitis B virus and hepatitis C virus infection, aflatoxin exposure, and alcoholic cirrhosis have been associated with the development of HCC, the genetic events involved in the pathogenesis of HCC are still unclear [3]. One of the most frequently detected genetic alterations in HCC is the amplification of the long arm of chromosome 1, which has been detected in 58–78% HCC patients by comparative genomic hybridization [4]–[7]. A minimal amplified region at 1q has been narrowed down to 1q21 [7], [8], suggesting the existence of an oncogene at 1q21 which plays an important role in HCC pathogenesis.

Recently, we used microdissected DNA from 1q21 to select region-specific transcripts from an HCC case with 1q21 amplification, and one candidate oncogene, named chromodomain helicase DNA binding protein 1-like gene (*CHD1L*, also called *ALC1*), was isolated [9]. *CHD1L* belongs to the SNF2-like family, containing a conserved SNF2_N domain, a helicase superfamily domain (HELICc) and a Macro domain. In our previous study, we found that *CHD1L* has strong oncogenic ability including increasing cell proliferation, colony formation in soft agar, and tumor formation in nude mice, and inhibiting tumor cell apoptosis [9]. To further investigate the *in vivo* oncogenic role of *CHD1L*, *CHD1L* ubiquitous-expression transgenic mouse model was generated and characterized in this study. Spontaneous tumors were found in 10 transgenic mice over 22 months of period. In addition, ethanol intoxication was found to promote the susceptibility of liver tumor formation in *CHD1L*-transgenic mice. The molecular mechanism of *CHD1L* in HCC development was also studied using mouse embryo fibroblasts (MEF).

## Results

### Generation of CHD1L transgenic mice

**Figure 1:**
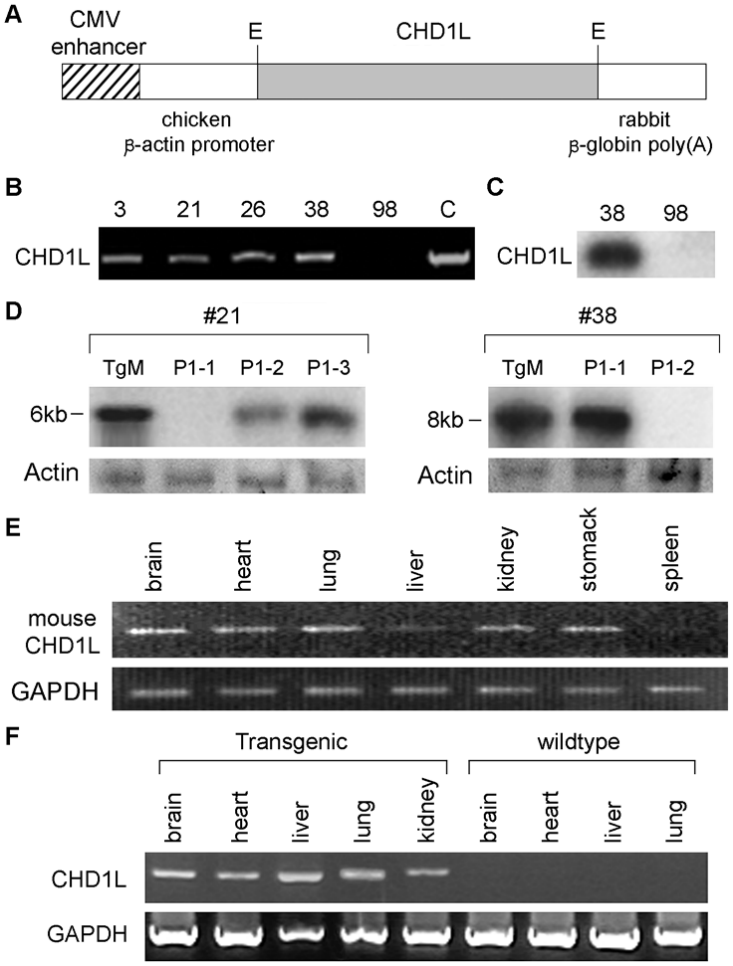
Generation of *CHD1L*-transgenic mouse model. (A) Construction of human *CHD1L* gene in pCAGGS for the generation of *CHD1L*-transgenic mouse. (B) Four *CHD1L*-transgenic mouse (#3, 21, 26, and 38) founders were identified by PCR screening. Genomic DNA from cloned CHD1L was used as positive controls. (C) CHD1L expression in transgenic mouse (#38) was confirmed by Northern blot analysis. (D) Two founders (lines 21 and 38) were able to transmit the transgene to their offspring (P1). Genomic DNA was digested with *Bam*HI and DNA fragment containing transgene *CHD1L* was detected by Southern blot analysis. The size of *Bam*HI-DNA fragment was 6 kb in line 21 and 8 kb in line 38, implying their integrated sites in host genomic DNA are different .

To further characterize the *in vivo* function of *CHD1L*, *CHD1L* ubiquitous-expression transgenic mouse model was generated. *CHD1L* was cloned into plasmid pCAGGS (**Fig. 1A**) and linearized constructs were injected into 773 F1 eggs and 40 pups were obtained. Among them, 4 independent founders including 3 males (lines 3, 26 and 38) and 1 female (line 21), were identified by PCR screening (**Fig. 1B**). Expression of CHD1L was confirmed by Northern blot analysis using a human specific CHD1L probe (Figure 1B). Two founders (lines 21 and 38) were capable of transmitting the transgene to their offspring and thus chosen for further functional studies (**Table 1** **,** **Fig. 1D** **)**. Two different *BamH*I fragments (6 kb in line 21 and 8 kb in line 38) containing transgene *CHD1L* were detected by Southern blot analysis, implying that *CHD1L* was integrated into different genomic sites in lines 21 and 38 (**Fig. 1D**).

**Table 1:** Summarization of *CHD1L*-transgenic founders and their offspring for tumorigenicity studies.

Founder	Sex	Offspring Tested	Germline Transmission Rate	Ethanol Intoxication^a^	Tumor Formation^b^
#3	male	52	2 (2.8%)		
#21	female	27	15 (55.6%)		27 (15)
#26	male	32	2 (6.25%)		
#38	male	65	32 (49.23%)	12 (6)	53 (26)

a Total number of offspring and *CHD1L*-transgenic offspring (in blanket) were used for ethanol intoxication study.

b Total number of offspring and *CHD1L*-transgenic offspring (in blanket) were used for spontaneous tumor formation study.

### Characterization of CHD1L-transgenic mice

**Figure 2:**
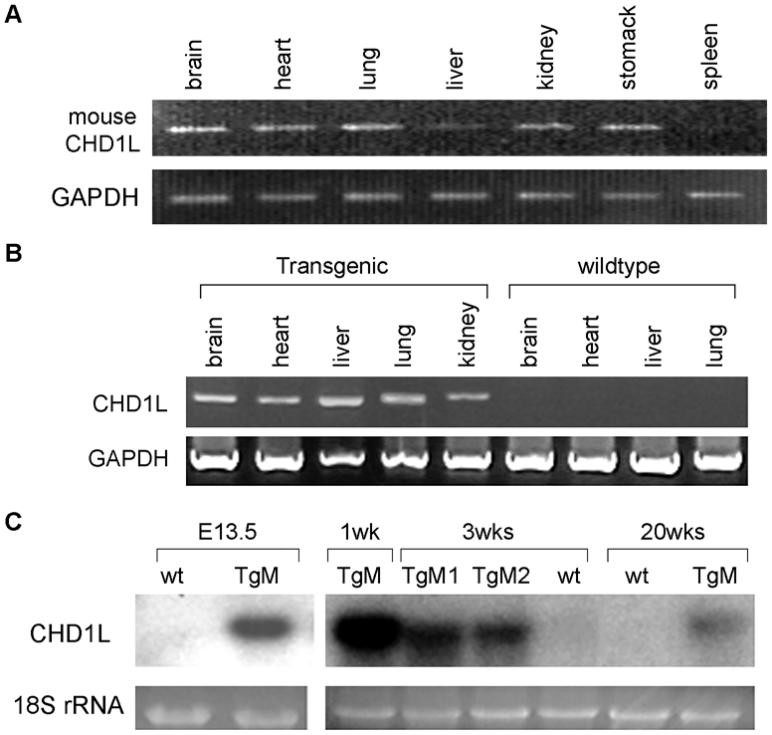
Characterization of CHD1L-transgenic mice. (A) Endogenous mouse CHD1L expression was tested by RT-PCR using mouse-specific primers. Weak expression of CHD1L was detected in liver and spleen. GAPDH was used as internal control. (B) Expression of transgene CHD1L in multiple tissues of transgenic and wildtype mice was studied by RT-PCR using human-specific primers in adult mice. GAPDH was used as internal control. (C) Expression of CHD1L in liver at different ages was tested by RT-PCR. 18S rRNA was used as loading control.

DNA sequence comparison showed that the homology between human and mouse *CHD1L* is about 81.2%. The endogenous expression of mouse CHD1L was studied by RT-PCR using mouse-specific primers and the result showed that high level expression of CHD1L was detected in brain, heart, lung, kidney and stomach. Low expression of endogenous mouse CHD1L was observed in liver and spleen (**Fig. 2A**). The expression of transgene CHD1L in CHD1L-transgenic and wildtype mice was tested by RT-CPR using human-specific primers. The results showed that expression of CHD1L was detected in all tested tissues (brain, heart, liver, lung, and kidney) in transgenic mice but not in wildtype mice (**Fig. 2B**). The expression of CHD1L in the liver of transgenic mice was firstly detected at embryonic stage 13.5dpc (**Fig. 2C**). The expression of CHD1L increased at 16dpc till 1 week after birth, and then decreased slightly at 3 to 20 weeks postnatal (**Fig. 2C**). *CHD1L*-transgenic mice were indistinguishable from wild-type siblings by size and weight.

### Ethanol intoxication promotes liver tumor susceptibility in transgenic mice

**Figure 3:**
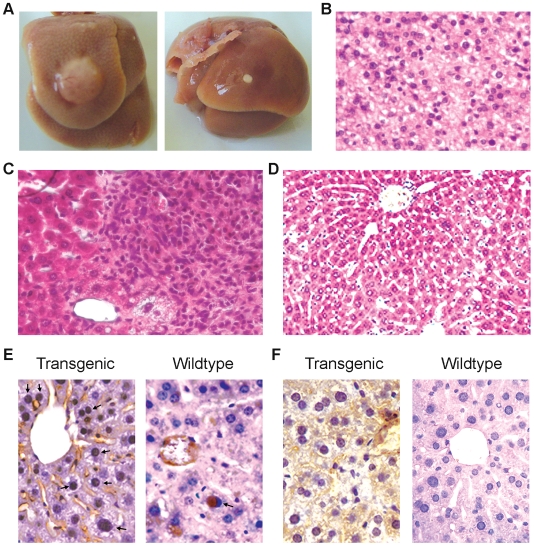
Ethanol intoxication promotes the susceptibility of liver tumor in *CHD1L*-transgenic mice. (A) A visible liver tumor (left) and adipoma (right) were found in *CHD1L*-transgenic mice following the ethanol exposure. (B) Histological study confirms the liver tumor is HCC. (C) Representative example of severe dysplasia lesion observed in a *CHD1L*- transgenic mouse following the ethanol exposure. (D) Representative example of normal liver tissue observed in a wildtype mouse following the ethanol exposure. (E) PCNA staining results showed that the frequency of cell proliferation (PCNA positive staining cells, indicated by arrows) was significantly higher in *CHD1L*-transgenic mice than that in wildtype littermates. (F) Representative example of AFP positive staining detected in a *CHD1L*- transgenic mouse and AFP negative staining in a wildtype mouse, respectively, after the ethanol exposure.

To investigate whether ethanol intoxication is able to promote the susceptibility of liver tumor in *CHD1L*-transgenic mice, wild type and *CHD1L*-transgenic mice (6 mice for each group) were exposed to ethanol intoxication for a period of 12 weeks. Liver pathology and the presence of a glossy appearance on the surface of the liver between the two groups of animals were compared. In *CHD1L*-transgenic mice, a visible liver solid tumor and an adipoma were observed in two different mice (**Fig. 3A**). Histology study confirmed that the liver tumor was HCC (**Fig. 3B**). Severe dysplasia lesion was observed in three other *CHD1L*-transgenic mice (**Fig. 3C**). No visible tumor and dysplasia lesion was detected in wildtype mice (**Fig. 3D**).

Following ethanol intoxication, the frequency of cell proliferation between *CHD1L*-transgenic and wildtype mice was compared by PCNA staining. The result revealed that the cell proliferation frequency was significantly higher in transgenic mice (5.5%) than that of wildtype littermates (1.6%, *p*<0.05, **Fig. 3E**). In addition, the expression of AFP can only be detected in *CHD1L*-transgenic mice but not in their wildtype littermates (**Fig. 3F**). Taken together, these observations suggest that overexpression of CHD1L in hepatocytes promotes the susceptibility of tumor formation in mouse.

### Spontaneous tumor formation in CHD1L-transgenic mice

**Figure 4:**
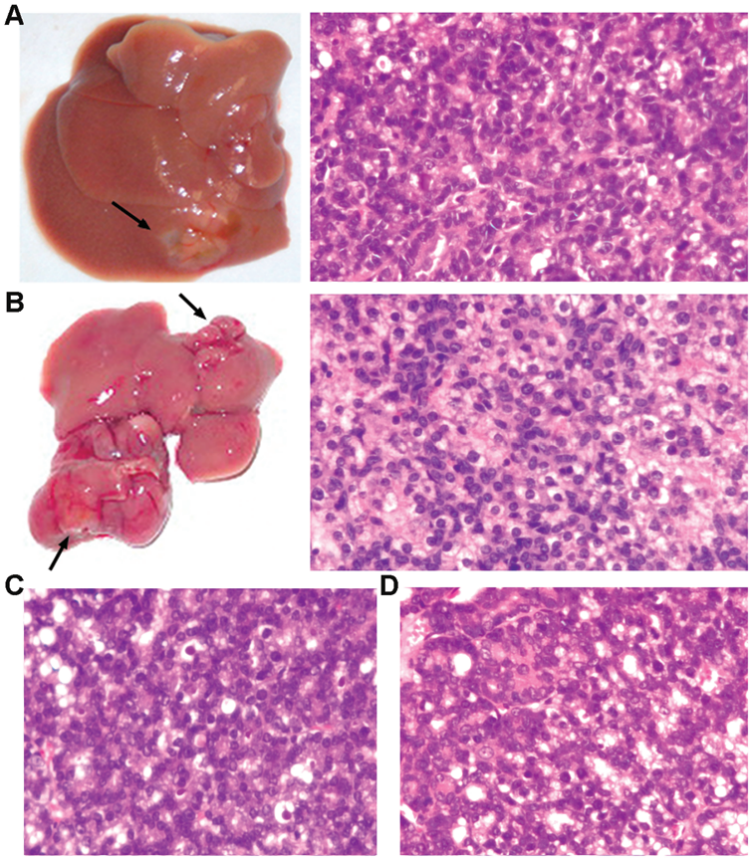
Detection of spontaneous liver tumors in *CHD1L*-transgenic mice. (A) Representative example of a visible liver tumor in one *CHD1L*-transgenic mouse (left), which was diagnosed as HCC by histological study (right). (B) Two liver tumors were found in one *CHD1L*-transgenic mouse (left, indicated by arrows) and histological study confirmed they are HCCs. (C and D) Representative examples of other two HCCs observed in *CHD1L*-transgenic mice.

**Figure 5:**
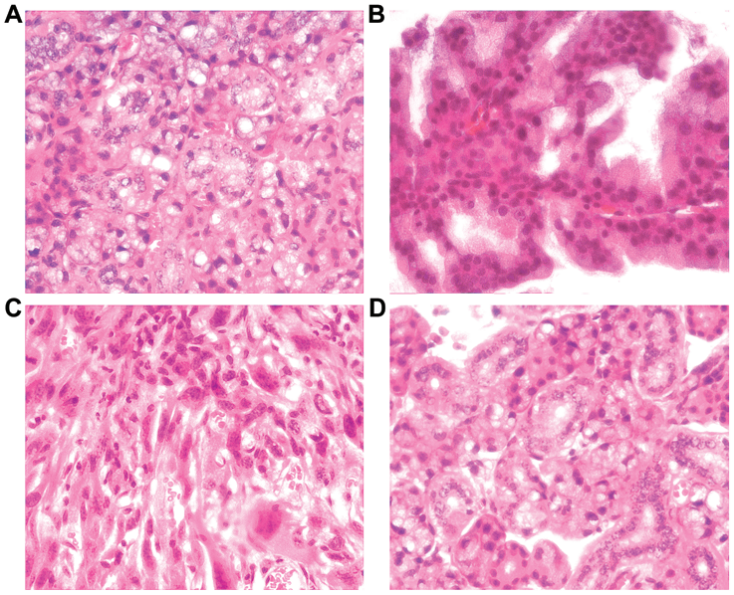
Detection of spontaneous tumors in other organs in CHD1L-transgenic mice. Besides HCC, several other different tumors were found in *CHD1L*-transgenic mice including salivary acinic cell adenocarcinoma (A and B), rhabdomyosarcoma (C), and colon adenocarcinoma (D).

Only first-generation offspring from founder lines #21 and #38 (41 *CHD1L*-transgenic mice and 39 wildtype mice) were used as study cohort. Spontaneous tumor formation was observed in 10/41 (24.4%) transgenic mice, but not in their 39 wild-type littermates over a monitoring period of 22 months. The places of tumor formed and their pathological diagnosis were summarized in Table 2. Liver tumors were observed in 4 mice and histological study revealed that they were all HCC (**Fig. 4**). In mouse 2, two liver tumors in similar size were found in different lobes (**Fig. 4B**, indicated by arrows). Two other spontaneous HCC tumors formed in *CHD1L*-transgenic mice were also shown in Figure 4 (4C and 4D). In mouse 5, two spontaneous tumors were found, one in the neck and the other in the uterus. Histological study revealed that they were salivary acinic cell adenocarcinoma (**Fig. 5A**) and uterine adenofibroma, respectively. In 10 CHD1L-transgenic mice suffered with tumor, different tumor types were found including HCC, salivary acinic cell adenocarcinoma, (**Figs. 5A and 5B**), rhabdomyosarcoma (**Fig. 5C**), gall bladder adenocarcinoma, and colon adenocarcinoma (**Fig. 5D** **).**

**Table 2:** Summarization of spontaneous tumor formation in *CHD1L*-transgenic mice.

Mouse^a^	Subcategory	Organ	Pathological Diagnosis
M1 (L21)	tumor 1	liver	HCC
	tumor 2	abdomen wall	adipoma
	tumor 3	abdomen wall	adipoma
M2 (L38)	tumor 1	liver	HCC
	tumor 2	liver	HCC
M3 (L38)	tumor 1	liver	HCC
M4 (L38)	tumor 1	liver	HCC
M5 (L21)	tumor 1	neck	salivary acinic cell adenocarcinoma
	tumor 2	uterus	uterine adenofibroma
M6 (L38)	tumor 1	face	salivary acinic cell adenocarcinoma
M7 (L38)	tumor 1	gall bladder	adenocarcinoma
M8 (L38)	tumor 1	colon	adenocarcinoma
M9 (L38)	tumor 1	posterior limb	rhabdomyosarcoma
M10 (L21)	tumor 1	abdomen wall	adipoma

a Mouse number was listed as M1 to M10. Offspring from founder #21 or #38 was shown in blanket.

### Overexpression of CHD1L promotes cell cycle in transgenic MEF

**Figure 6:**
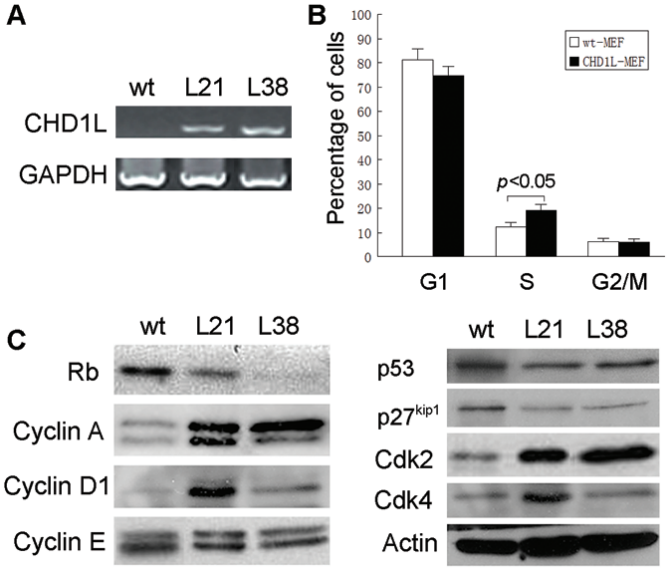
Overexpression of CHD1L promotes cell proliferation in *CHD1L*-transgenic MEF. (A) Detection of CHD1L expression in MEFs from *CHD1L*-transgenic (Tg) and wild type (Wt) mice by RT-PCR. GAPDH was used as internal control. (B) Examples of DNA content in *CHD1L*-transgenic and wild type MEFs detected by flow cytometry. (C) Western blot analyses indicated that Cyclin D1, Cyclin A and CDK2, 4 were up-regulated, whiles p53, Rb and p27^Kip1^ were down-regulated in *CHD1L*-transgenic MEFs compared with their wild type MEFs (pool of two MEFs). β-actin was used as loading control.

Mouse embryonic fibroblasts (MEF) derived from *CHD1L*-transgenic mice and their wildtype littermates were established using embryos at 13.5dpc. Pool of two wildtype MEFs and two *CHD1L*-transgenic MEFs were tested and their CHD1L expression level was determined by RT-PCR (**Fig. 6A**). DNA content analysis with MEF cells by flow cytometry found that CHD1L could facilitate DNA synthesis and G1/S transition (**Fig. 6B**). To address the possible pathway involved, expressions of several cell cycle-related genes, including Rb, Cyclin A, Cyclin D1, Cyclin E, CDK2, CDK4, p27^kip1^, and p53 were compared between wildtype and *CHD1L*-transgenic MEFs. The results showed that expressions of Cyclin A, Cyclin D1, CDK2, and CDK4 were notably up-regulated and a slight increase in CyclinE expression level, while Rb, p27^kip1^ and p53 were down-regulated in *CHD1L*-transgenic MEFs compared with wildtype MEFs (**Fig. 6C**).

## Discussion

Amplification of 1q21 is one of the most frequently detected genetic changes in HCC. Activation of oncogene plays critical role in cancer development and one common mechanism of oncogene activation is overexpression caused by DNA amplification. Our previous study showed that amplification of 1q21 is an early event in HCC development, implying that the putative oncogene within this region may play important role in the initiation of HCC pathogenesis [10]. Recently, one candidate oncogene, *CHD1L*, was isolated from 1q21 and our previous study has demonstrated its oncogenic ability. In this report, we described the generation and characterization of a *CHD1L*-transgenic mouse model.

*CHD1L* transgenic mouse model established in this study provides a very useful tool for investigating *CHD1L* function and role in cancer development. Two founders (#21 and #38) were capable of transmissing the transgene *CHD1L* to their offspring and Southern blot analysis found that their integrating sites are different. Therefore, these two lines were selected for further study. Spontaneous tumor formation was found only in old *CHD1L*-transgenic mice (over 20 months old), implying that the initiation and progression of HCC carcinogenesis caused by abnormal *CHD1L* expression is a long process. Several different types of tumors were found in *CHD1L*-transgenic mice including HCC, salivary, colon, uterine, and gall bladder adenocarcinoma, and rhabdomyosarcoma. Amplification of 1q has been also frequently detected in other solid tumors, including colon cancer [11], uterine cancer [12], and rhabdomyosarcoma [13]. These data suggest that *CHD1L* may play an important role in cancer development in many solid tumors.

To shorten the latency of tumor onset, alcohol intoxication was employed to induce hepatocyte pathological lesions. Alcohol is one the major risk factor for hepatocellular carcinoma, especially in western countries. Alcohol intoxication is a simple but efficient way to induce liver lesions in cancer-prone marine [14]. Interestingly, after 12 weeks alcohol treatment, 5/6 of *CHD1L*-transgenic mice had liver lesions, including HCC, adipoma, and severe dysplasia. However, no obvious precancerous lesion was detected in wildtype controls. In addition, overexpression of CHD1L could increase hepatocyte proliferation and induce AFP expression in transgenic liver post alcohol treatment. These results suggest that overexpression of CHD1L in liver increases the tumor susceptibility.

Promotion of cell proliferation is a major molecular mechanism of oncogene in cancer development. In this study, we demonstrated that *CHD1L* could facilitate DNA synthesis and promoted G1/S phase transition in *CHD1L*-transgenic MEFs. This result is consistent with our previous finding, in which overexpression of CHD1L in QGY-7703 (HCC cell line) cells could also promote G1/S phase transition [9]. To further explore the molecular mechanism of *CHD1L* in cell cycle promotion, expressions of several G1/S phase transition checkpoint related proteins were compared between *CHD1L*-transgenic and wildtype MEFs. The results showed that *CHD1L* could up-regulate Cyclin A, Cyclin D1, CDK2 and CDK4, and down-regulate Rb, p27^Kip1^ and p53. p27^Kip1^ serves as a key mediator in G1/S transition through Cdk inhibition and regulating the activity of cyclin D-Cdk4/6 complex which are essential for S phase entry [15], [16]. The reduced expression of p27^Kip1^ facilitates the activation of cyclin D-Cdk4/6 complex, resulting in the cyclin D-Cdk4/6-medicated Rb phosphorylation and destruction of Rb-E2F binding. The releasing E2F activates the transcription of genes necessary for S phase entry and progression [17]. Overexpression of CHD1L might serve as mitogenic signal to induce expression of cyclin D-Cdk4/6 and inhibit the expression of p27^Kip1^. In addition, the p53 protein is a transcription factor that upregulates the expression of p21^Waf1/Cip1^, which in turn functions as a Cdk2 inhibitor to control S phase entry via the inactivation of cyclinE-Cdk2 complex. The notable upregulation of Cdk2 in *CHD1L*-transgenic MEFs suggested that the dysregulation of the p53–cyclinE-Cdk2 pathway might be also involved in CHD1L-induced G1/S transition. Taken together, the observations in the present study strongly support that *CHD1L* is a novel oncogene responsible for the 1q21 amplification event in HCC and plays an important role in the development of HCC via promoting cell cycle.

## Materials and Methods

### Generation of CHD1L-transgenic mice

For construction of *CHD1L*-transgenic mice, a 2.8-kb *EcoR*I–*EcoR*I fragment containing the open reading frame of human *CHD1L* gene (Gene Bank accession no. AF537213), which was amplified by PCR from normal human liver cell line LO-2 complementary DNA, was cloned into plasmid pCAGGS. Expression of CHD1L was driven by a human CMV-IE enhancer linked to the chicken β-actin promoter, followed by its first exom and intron (**Fig. 1A**). The human *CHD1L* has its own stop codon followed by a rabbit β-globin poly(A) sequence. The mRNA transcript of the transgene consists of the first exon of chicken β-actin, which is transcribed but not translated, followed by the *CHD1L* transgene. The generation of *CHD1L*-transgenic mice was performed using the standard method [18]. Briefly, the linearized constructs were injected into one-cell-stage F1 eggs (DBA×C57BL/6), which were transplanted into pseudo-pregnant females (average 12 eggs per oviduct).

All resulting pups were screened for the presence of the transgene by PCR using genomic DNA obtained from tail snips with a pair of human-specific primers (Forward: 5′-AGCGCCTGGCTTCTTACTGC; Reverse: 5′-GCTTATCCAGCAGGTGAAGCTTC). The *CHD1L*-transgenic founders were crossed with wildtype F1 (DBA/C57bl6) and their first-generation offsprings (47 *CHD1L*-transgenic mice and 45 wildtype mice) were used as study cohort (**Table 1**). Mouse endogenous CHD1L expression was tested by RT-PCR using a pair of mouse-specific primers (Forward: 5′-GGAGGAGGAAGCCTAGAACC; Reverse: 5′-CGCTGCTTCCTGTCTTTTCT). Animal experimentation was done according to the institutional guidelines (Association for Assessment and Accreditation of Laboratory Animal Care International) for animal care. All the animal experiments were approved by the Committee on the Use of Live Animals in Teaching and Research (CULATR), The University of Hong Kong.

### Southern and Northern blot analyses

For Southern blot analysis, 10 µg of genomic DNA was digested with *BamH*I, fractionated on 1% agarose gel, transferred to a nylon membrane, and hybridized overnight at 42°C with ^32^P-labeled probe for human *CHD1L* gene. For Northern blot analysis, 20 µg of total cellular RNA was size fractionated, transferred to a nylon membrane, and hybridized with ^32^P-labeled human *CHD1L* gene.

### Ethanol intoxication

*CHD1L*-transgenic mice and their wild type littermates (6 mice for each group) at age of 20-weeks old were fed *ad libitum* a mixture containing increasing concentrations of ethanol (10% for 2 days; 15% for 3 days and thereafter 20%) in 20% sucrose during the entire treatment period (12 weeks). This mixture was the only source of drinking fluid for the animal for the entire duration of the experiment. Animals were then kept without ethanol for 4 weeks before sacrificed.

### Histological and pathological study

Animals with visible tumors were sacrificed when signs of distress appeared. Tumors were immediately removed and fixed in 10% formalin for 24 hr. After dehydration, the tumor tissues were paraffin embedded. Serial sections (5 µm in thickness) were prepared, stained with Mayer's hematoxylin-eosin (H&E), and examined under microscope by two independent pathologists. Immunohistochemistry (IHC) was performed using standard streptavidin-biotin-peroxidase complex method with anti PCNA and AFP (Santa Cruz Biotechnology, Santa Cruz, CA) antibodies.

### Generation of mouse embryo fibroblast (MEF)

Pregnant female mouse at 13.5 dpc was sacrificed and embryos were collected. The embryos were minced thoroughly with sterile scissors and then digested in 0.2% typsin (Sigma, St. Louis, MO) at 37°C for 10 min. The cell suspension was cultured in Dulbecco's modified Eagle's medium (DMEM) supplemented with 10% FBS at 37°C.

### Detection of DNA content by flow cytometry

DNA content in *CHD1L*-transgenic MEF and wildtype MEF was compared by flow cytometry. Cells were fixed in 70% ethanol for at least 1 hr and stained with staining solution (100 µg/ml RNase, 0.02% Triton X-100, 10 µg/ml propidium iodide) for 1 hr. Samples were analyzed using FACSCalibur flow cytometer and CellQuest software (BD Biosciences, San Jose, CA).

### Western blotting analysis

For Western blotting, 20 µg of protein extract was separated by SDS–polyacrylamide gel electrophoresis and transferred to a PVDF Hybond-P membrane (Amersham Pharmacia Biotechnology, Piscataway, NJ). Western blot analyses were performed by a standard method with antibodies to Rb, Cyclin D1, Cyclin A, Cyclin E, CDK2, CDK4, p27^kip1^, p53 and β-Actin (Cell Signaling Technology, Beverley, MA).

### Statistical analysis

The difference of PCNA positive cells in *CHD1L-*transgenic liver and wildtype counterparts was compared with Student's T-test. The difference of DNA content between *CHD1L*-transgenic and wildtype MEFs was compared with Student's T-test. *P* values of <0.05 were considered to be significant.

## References

[1] Llovet JM, Burroughs A, Bruix J (2003). Hepatocellular carcinoma.. Lancet.

[2] El-Serag HB, Mason AC, Key C (2001). Trends in survival of patients with hepatocellular carcinoma between 1977 and 1996 in the United States.. Hepatology.

[3] Montalto G, Gervello M, Giannitrapani L, Dantona F, Terranova A (2002). Epidemiology, risk factors, and natural history of hepatocellular carcinoma.. Ann NY Acad Sci.

[4] Marchio A, Meddeb M, Pineau P, Danglot G, Tiollais P (1997). Recurrent chromosomal abnormalities in hepatocellular carcinoma detected by comparative genomic hybridization.. Genes Chromosomes Cancer.

[5] Kusano N, Shiraishi K, Kubo K, Oga A, Okita K (1999). Genetic aberrations detected by comparative genomic hybridization in hepatocellular carcinomas: their relationship to clinicopathological features.. Hepatology.

[6] Wong N, Lai P, Lee SW, Fan S, Pang E (1999). Assessment of genetic changes in hepatocellular carcinoma by comparative genomic hybridization analysis: relationship to disease stage, tumor size, and cirrhosis.. Am J Pathol.

[7] Guan XY, Fang Y, Sham JS, Kwong DL, Zhang Y (2000). Recurrent chromosome alterations in hepatocellular carcinoma detected by comparative genomic hybridization.. Genes Chromosomes Cancer.

[8] Qin LX, Tang ZY, Sham JS, Ma ZC, Ye SL (1999). The association of chromosome 8p deletion and tumor metastasis in human hepatocellular carcinoma.. Cancer Res.

[9] Ma NF, Hu L, Fung JM, Xie D, Zheng BJ (2008). Isolation and characterization of a novel oncogene, amplified in liver cancer 1, within a commonly amplified region at 1q21 in hepatocellular carcinoma.. Hepatology.

[10] Wang Y, Wu MC, Sham JST, Zhang W, Wu WQ (2002). Prognostic significance of c-myc and AIB1 amplification in hepatocellular carcinoma. A broad survey using high-throughput tissue microarray.. Cancer.

[11] He QJ, Zeng WF, Sham JST, Xie D, Yang XW (2003). Recurrent genetic alterations in 26 colorectal carcinomas and 21 adenomas from Chinese patients.. Cancer Genet Cytogenet.

[12] Schulten HJ, Gunawan B, Enders C, Donhuijsen K, Emons G (2004). Overrepresentation of 8q in carcinosarcomas and endometrial adenocarcinomas.. Am J Clin Pathol.

[13] Kiuru-Kuhlefelt S, El-Rifai W, Sarlomo-Rikala M, Knuutila S, Miettinen M (1998). DNA copy number changes in alveolar soft part sarcoma: a comparative genomic hybridization study.. Mod Pathol.

[14] Pani G, Fusco S, Colavitti R, Borrello S, Maggiano N (2004). Abrogation of hepatocyte apoptosis and early appearance of liver dysplasia in ethanol-fed p53-deficient mice.. Biochem Biophys Res Commun.

[15] Sherr CJ, Roberts JM (1999). CDK inhibitors: positive and negative regulators of G1-phase progression.. Genes Dev.

[16] Vogelstein B, Lane D, Levine AJ (2000). Surfing the p53 network.. Nature.

[17] Harbour JW, Luo RX, DeiSanti A, Postigo AA, Dean DC (1999). Cdk phosphorylation triggers sequential intranmolecular interactions that progressively block Rb functions as cells move through G1.. Cell.

[18] Hogan B (1983). Molecular biology. Enhancers, chromosome position effects, and transgenic mice.. Nature.

